# Bone Resorption and Environmental Exposure to Cadmium in Women: A Population Study

**DOI:** 10.1289/ehp.11167

**Published:** 2008-02-25

**Authors:** Rudolph Schutte, Tim S. Nawrot, Tom Richart, Lutgarde Thijs, Dirk Vanderschueren, Tatiana Kuznetsova, Etienne Van Hecke, Harry A. Roels, Jan A. Staessen

**Affiliations:** 1 Studies Coordinating Centre, Division of Hypertension and Cardiovascular Rehabilitation Unit, Department of Molecular and Cardiovascular Research, University of Leuven, Leuven, Belgium; 2 Department of Physiology, School for Physiology, Nutrition and Consumer Sciences, North-West University, Potchefstroom, South Africa; 3 Section of Experimental Medicine and Endocrinology, Department of Experimental Medicine, University of Leuven, Leuven, Belgium; 4 Section of Social and Economic Geography, Department of Geography and Geology, University of Leuven, Leuven, Belgium; 5 Industrial Toxicology and Occupational Medicine Unit, Department of Public Health, Université catholique de Louvain, Brussels, Belgium

**Keywords:** bone, cadmium, pyridinium crosslinks

## Abstract

**Background:**

Environmental exposure to cadmium decreases bone density indirectly through hypercalciuria resulting from renal tubular dysfunction.

**Objective:**

We sought evidence for a direct osteotoxic effect of cadmium in women.

**Methods:**

We randomly recruited 294 women (mean age, 49.2 years) from a Flemish population with environmental cadmium exposure. We measured 24-hr urinary cadmium and blood cadmium as indexes of lifetime and recent exposure, respectively. We assessed the multivariate-adjusted association of exposure with specific markers of bone resorption, urinary hydroxylysylpyridinoline (HP) and lysylpyridinoline (LP), as well as with calcium excretion, various calciotropic hormones, and forearm bone density.

**Results:**

In all women, the effect sizes associated with a doubling of lifetime exposure were 8.4% (*p* = 0.009) for HP, 6.9% (*p* = 0.10) for LP, 0.77 mmol/day (*p* = 0.003) for urinary calcium, –0.009 g/cm^2^ (*p* = 0.055) for proximal forearm bone density, and –16.8% (*p* = 0.065) for serum parathyroid hormone. In 144 postmenopausal women, the corresponding effect sizes were –0.01223 g/cm^2^ (*p* = 0.008) for distal forearm bone density, 4.7% (*p* = 0.064) for serum calcitonin, and 10.2% for bone-specific alkaline phosphatase. In all women, the effect sizes associated with a doubling of recent exposure were 7.2% (*p* = 0.001) for urinary HP, 7.2% (*p* = 0.021) for urinary LP, –9.0% (*p* = 0.097) for serum parathyroid hormone, and 5.5% (*p* = 0.008) for serum calcitonin. Only one woman had renal tubular dysfunction (urinary retinol-binding protein > 338 μg/day).

**Conclusions:**

In the absence of renal tubular dysfunction, environmental exposure to cadmium increases bone resorption in women, suggesting a direct osteotoxic effect with increased calciuria and reactive changes in calciotropic hormones.

Cadmium is a persistent environmental toxicant (Hogervorst et al. 2007; Lauwerys and Hoet 2001). Sources of cadmium pollution are past and present emissions from non-ferrous industries, waste incineration, use of cadmium-containing phosphate fertilizers and sewage sludge, and the burning of fossil fuels (Lauwerys and Hoet 2001). Human exposure to cadmium occurs through consumption of contaminated food or water (Hogervorst et al. 2007; Watanabe et al. 2004) or by inhalation of tobacco smoke or polluted air (Hogervorst et al. 2007). Cadmium accumulates in the human body, in particular in the liver and kidneys, and has an elimination half-life of 10–30 years (Järup et al. 1998). The urinary excretion of cadmium over 24 hr is a bio-marker of lifetime exposure (Järup et al. 1998). Cadmium causes glomerular and tubular renal dysfunction (Staessen et al. 1994) and increases calciuria (Staessen et al. 1991).

Current estimates suggest that > 200 million people worldwide have osteoporosis and that the prevalence of this disease is escalating (Reginster and Burlet 2006). Staessen et al. (1999) showed that low-level environmental cadmium exposure promotes osteoporosis and leads to a higher risk of fractures, especially in postmenopausal women. Women are at greater risk of developing cadmium toxicity than are men (Choudhury et al. 2001). Animal (Wang et al. 1994) and *in vitro* (Regunathan et al. 2003) studies suggest that cadmium might have direct toxic effects on bone, but convincing evidence for such an effect in humans does not exist. The development of biochemical assays that measure pyridinium crosslinks of collagen, which are specific markers of bone resorption (McLaren et al. 1992), greatly facilitates the exploration of cadmium’s osteotoxicity. In view of the epidemic of osteoporosis (Reginster and Burlet 2006) and the ubiquitous distribution of cadmium pollution (Lauwerys and Hoet 2001), we used urinary crosslinks as a marker to investigate the possible direct osteotoxicity of cadmium (over and beyond its indirect effects on bone via increased calciuria) (Staessen et al. 1991) in Flemish women living in districts with low to moderate environmental cadmium pollution.

## Methods

### Fieldwork

The Cadmium in Belgium study (CadmiBel, 1985–1989) (Lauwerys et al. 1990) included 1,107 Flemish participants randomly recruited from 10 districts in northeastern Belgium (Staessen et al. 1994). The participation rate was 78% (Staessen et al. 1994). The geometric mean cadmium concentration in the soil sampled from 85 kitchen gardens was 5.3 mg/kg (5th–95th percentile interval, 1.4–18.9) in six districts, which bordered on three zinc/cadmium smelters, and 0.9 mg/kg (0.4–1.6) in four districts, which were > 10 km away from the smelters (Hogervorst et al. 2007). The participants of the 10 districts had similar characteristics apart from exposure to cadmium (Staessen et al. 1994, 1999). We complied with all applicable requirements of U.S. and international regulations, in particular the Helsinki declaration for investigation of human subjects. The Ethics Review Board of the Medical Faculty of the University of Leuven approved the study. Participants gave informed consent at recruitment.

From 1991 to 1996, in the framework of the Public Health and Environmental Exposure to Cadmium study (PheeCad), we invited 823 former CadmiBel participants, who had renewed their informed consent, for a measurement of bone density, of whom 614 (74.6%) responded. This cohort included 307 women, whose exposure to cadmium was exclusively environmental. Because of missing information, we excluded 13 women. Thus, the study population for the present analysis consisted of 294 women.

### Clinical measurements

As described elsewhere (Staessen et al. 1999), from 1991 to 1996 we measured bone density at the forearm just above the wrist by single photon absorptiometry (ND1100 bone density scanner; Nuclear Data Inc, Schaumburg, IL, USA). Distal scans of adult forearms traverse a mean of 35% trabecular bone, whereas in proximal scans this proportion declines to nearly 5%. Trained nurses measured the anthropometric characteristics of the women. They administered a questionnaire to collect information about the participants’ lifestyle and medication intake. Socioeconomic status was coded and condensed into a scale with scores ranging from 1 to 3. Using published tables, we computed the energy spent in physical activity from body weight, time devoted to work and sports, and type of physical activity. Premenopause was defined as an active menstrual cycle throughout follow-up. Menopause was defined as the absence or cessation of periods during follow-up, confirmed by measurement of the serum concentration of follicle-stimulating hormone (FSH).

### Biochemical measurements

At baseline (1985–1989) and follow-up (1991–1996), the participants collected a 24-hr urine sample in a wide-neck polyethylene container for the measurement of cadmium, calcium, retinol-binding protein, and creatinine. For these measurements, we applied the same analytical methods throughout the study. We measured serum and urinary creatinine (Bartels and Böhmer 1971) using an automated enzymatic technique (Technicon Autoanalyzer, Technicon Instruments, Tarrytown, NY, USA). At follow-up (1991–1996), the nurses obtained a venous blood sample from the participants within 2 weeks of the bone density measurement. We measured the serum concentration of FSH (AR FSH Reagent Kit, Abbott 6C24-20; Louvain-la-Neuve, Belgium), calcitonin (CT-U.S.-IRMA Kit, BioSource Europe, Nivelles, Belgium ), and γ-glutamyltransferase, an index of alcohol intake, using commercially available kits. We determined bone-specific serum alkaline phosphatase activity on a COBAS-BIO centrifugal analyzer (Roche Diagnostics, Vilvoorde, Belgium), serum parathyroid hormone (PTH) by a two-site immunometric assay (Bouillon et al. 1990), serum and urinary calcium by compleximetry, and urinary retinolbinding protein, a biomarker of renal tubular dysfunction, by an automated nonisotopic immunoassay based on latex particle agglutination (Lauwerys et al. 1990).

To measure blood and urinary cadmium, we applied electrothermal atomic absorption spectrometry with a stabilized-temperature platform furnace and Zeeman background correction (Lauwerys et al. 1990). The external quality-control program did not show any time trend in the accuracy of the cadmium measurements. In the context of this article, we used the average of the 24-hr urinary cadmium excretion at baseline (1985–1989) and follow-up (1991–1996) as a measure of lifetime exposure and the blood cadmium concentration at the time of the urine collection for crosslinks (1991–1996) as a biomarker of recent exposure.

On the day of the bone density measurement (1991–1996), the participants collected an exactly timed 4-hr urine sample for the measurement of the collagen pyridinium crosslinks, hydroxylysylpyridinoline (HP) and lysylpyridinoline (LP), and creatinine. We measured HP and LP by high-performance liquid chromatography, using a slight modification of the methods described by Black et al. (1998) and Uebelhart et al. (1990). The intra- and interassay precision was 5.9% and 7.0% for HP, and 9.5% and 10.2% for LP. The sensitivity of the assay was 1 pmol.

### Statistical analyses

For database management and statistical analysis, we used SAS software (version 9.1; SAS Institute Inc., Cary, NC, USA). We logarithmically transformed variables with a non-Gaussian distribution. We represented the central tendency and spread of transformed variables by the geometric mean and the 5th to 95th percentile interval. We compared means and proportions by use of the large sample *z*-test and the chi-square statistic, respectively. We assessed longitudinal changes in proportions by McNemar’s test. We compared parity across subgroups of women by Kruskall–Wallis test. All *p*-values refer to two-sided hypotheses.

We plotted mean values of the biomarkers of effect by quartiles of the exposure measures to ensure that there was no threshold phenomenon and that linear correlation techniques were appropriate. We investigated associations between biomarkers of effect and exposure using single and multiple linear regressions. We identified covariates by a stepwise regression procedure with the *p*-values for variables to enter and to stay in the model set at 0.15. Covariates considered for entry in the model were age, age squared, body mass index, smoking, γ-glutamyl-transferase activity in serum, intake of diuretics, use of supplements of calcium and/or vitamin D, menopausal status, hormone replacement therapy and/or oral contraception, parity, energy spent in physical activity, socioeconomic status, and urinary retinol-binding protein.

The 5th–95th percentile interval of the blood cadmium concentration and the 24-hr urinary cadmium excretion spanned approximately a 10-fold increase. Because we had normalized the distributions of blood and urinary cadmium by a logarithmic transformation, we expressed changes in the biomarkers of effect as the effect sizes related to a doubling of blood or urinary cadmium. We estimated these responses and their 95% confidence interval (CI) by multiplying regression coefficients (± 1.96 × SE) by 0.3 (the logarithm of 2).

## Results

### Characteristics of women

The median interval between baseline and the follow-up examination was 6.6 years (range, 5.3–10.5 years). Based on the questionnaires administered at baseline and follow-up and the measurement of FSH in serum at follow-up, the study population included 150 premenopausal and 144 menopausal women. Table 1 lists the women’s characteristics by menopausal status. Age at enrollment ranged from 20.4 to 73.6 years. As expected, bone density was lower in menopausal than in premenopausal women, whereas the opposite was true for the urinary excretion of crosslinks. The median time at which the participants started collecting urine for the measurement of crosslinks was 1000 hours (interquartile range, 0700–1200 hours).

From baseline to follow-up, blood cadmium decreased by 29.5% (95% CI, 24.5 to 34.2%; *p* < 0.0001), and 24-hr urinary cadmium declined by 12.7% (95% CI, 8.0 to 17.2%; *p* < 0.0001). During follow-up, the number of women who smoked decreased (*p* = 0.003) from 103 (35.0%) to 86 (29.2%). In smokers, the median daily tobacco use was 15 cigarettes (5th–95th percentile interval, 4–30 cigarettes). During follow-up, no significant changes occurred in the prevalence of alcohol consumption [15 (5.1%) vs. 22 (7.5%) women; *p =* 0.13], the energy spent in physical activity (454 vs. 442 kcal/day; *p* = 0.90), the intake of oral contraceptives [43 (14.6%) vs. 42 (14.3%); *p* = 0.87] or hormone replacement therapy [3 (1.0%) vs. 7 (2.4%); *p* = 0.16]. More women used diuretics at the follow-up examination than at baseline [34 (11.6%)] vs. 25 (8.5%); *p* = 0.029].

### Unadjusted analyses

**Figure 1:**
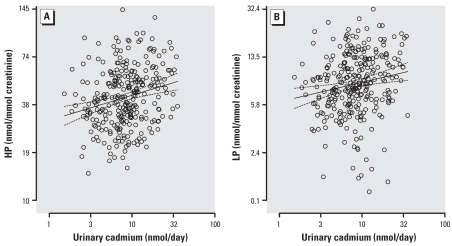
The urinary excretion of pyridinium crosslinks as a function of the 24-hr urinary cadmium excretion in 294 women in single regression analysis for (*A*) HP (*r* = 0.23; *p* < 0.0001) and (*B*) LP (*r* = 0.17; *p* = 0.003). The 24-hr urinary cadmium excretion was the average of two urine collections at a median interval of 6.6 years and reflects lifetime exposure. Solid and dashed lines represent the regression line and the 95% CI boundaries, respectively.

In single regression analysis, we noticed positive correlations of the urinary excretion of HP (*r* = 0.23; *p* < 0.0001) and LP (*r* = 0.17; *p* = 0.003) with the 24-hr excretion of cadmium (Figure 1), which was mirrored by inverse associations of proximal (–0.32; *p* < 0.0001) and distal (–0.22; *p* = 0.0001) forearm bone density with the biomarker of lifetime exposure. In addition, there was a positive correlation between the 24-hr urinary excretion of calcium and cadmium (*r* = 0.19, *p* = 0.0009).

### Adjusted analyses

In exploratory analyses, we studied the associations of biomarkers of effect with the 24-hr cadmium excretion across quartiles with adjustments applied for age, age squared, and menopausal status (Table 2). Urinary HP excretion increased significantly with higher 24-hr cadmium excretion (*p* for trend, 0.028). LP showed the same tendency (*p* for difference between the lowest and highest quartile, 0.095). The 24-hr calciuria increased significantly with higher 24-hr urinary cadmium (*p* for trend, 0.0002), with an opposite trend for PTH (*p* for difference between the lowest and highest quartile, 0.046) (Table 2). In the minimally adjusted analyses across quartiles of 24-hr urinary cadmium excretion, the trends in the serum levels of total calcium, bone-specific alkaline phosphatase, and calcitonin did not reach significance.

The independent associations between the effect biomarkers and the index of lifetime exposure are shown in Tables 3 and 4. With adjustments applied for the significant covariates, which were identified by stepwise regression, a doubling of the urinary cadmium excretion was associated with increases in the urinary excretion of HP and LP, and in 24-hr urinary calcium amounting to 8.4% (95% CI, 2.1 to 15.0%; *p* = 0.009), 6.9% (95% CI, –1.9 to 16.4%; *p* = 0.10), and 0.77 mmol/day (95% CI, 0.27 to 1.27 mmol/day; *p* = 0.003), respectively. In all women, doubling of urinary cadmium excretion was associated with small decreases in the proximal bone density (–0.00903 g/cm^2^; 95% CI, –0.00014 to 0.01819 g/cm^2^; *p* = 0.055) and in the serum concentration of PTH (–16.8%; 95% CI, –37.5 to 0.9%; *p* = 0.065). In menopausal women, the effect sizes associated with a doubling of 24-hr urinary cadmium were –0.01223 g/cm^2^ (95% CI, –0.00322 to –0.02123 g/cm^2^; *p* = 0.008) for the distal forearm bone density, 4.7% (95% CI, –0.3 to 9.9%; *p* = 0.064) for serum calcitonin, and 10.2% (95% CI, 1.4 to 19.7%; *p* = 0.023) for the activity in serum of bone-specific alkaline phosphatase.

The independent associations between the effect biomarkers and the index of current exposure appear in Tables 5 and 6. For each effect biomarker, we adjusted these relations for the same covariates as in Tables 3 and 4. In all women, the effect sizes associated with a doubling of blood cadmium concentration were 7.2% (95% CI, 2.9 to 11.6%; *p* = 0.001) for urinary HP, 7.2% (95% CI, 1.1 to 13.7%; *p* = 0.021) for urinary LP, –9.0% (95% CI, –18.5 to 1.7%; *p* = 0.097) for serum PTH, and 5.5% (95% CI, 1.5 to 9.8%; *p* = 0.008) for serum calcitonin. In postmenopausal women, doubling of the blood cadmium concentration was associated with a decrease in distal forearm bone density and an increase in bone-specific alkaline phosphatase activity. The effect sizes were –0.00870 g/cm^2^ (95% CI, –0.01693 to –0.00047 g/cm^2^; *p* = 0.038) and 7.0% (95% CI, –1.1 to 15.7%; *p* = 0.091).

### Sensitivity analyses

In all women but one, the urinary excretion of retinol-binding protein was below the cut-off value for early renal tubular dysfunction (≥ 338 μg/day) (Buchet et al. 1990). The urinary excretion of retinol-binding protein, socioeconomic status, the use of food supplements containing calcium and/or vitamin D, and parity did not enter any regression model. After forcing these four additional independent variables into our regression models, our findings remained consistent. In all women, the effect sizes associated with a doubling of 24-hr urinary cadmium were 8.2% (95% CI, 1.7 to 15.1%; *p* = 0.013) for HP, 6.5% (95% CI, –2.5 to 16.3%; *p* = 0.16) for urinary LP, 0.87 mmol/day (95% CI, 0.35 to 1.38 mmol/day; *p* = 0.001) for 24-hr urinary calcium, and –19.7% (95% CI, –41.5 to –1.3%; *p* = 0.035) for serum PTH. In all women, the estimates associated with a doubling of blood cadmium were 7.3% (95% CI, 2.9 to 11.9%; *p* = 0.001) for HP, 7.6% (95% CI, 1.2 to 14.3%; *p* = 0.019) for LP, –0.3% (95% CI, –7.1 to 7.4%; *p* = 0.96) for serum PTH, and 5.9% (95% CI, 1.7 to 10.2%; *p* = 0.006) for serum calcitonin. With these additional adjustments applied, in menopausal women, distal forearm bone density decreased by 0.01241 g/cm^2^ (95% CI, 0.00335 to 0.02147 g/cm^2^; *p* = 0.008) and by 0.00902 g/cm^2^ (95% CI, 0.00053 to 0.01752 g/cm^2^; *p* = 0.038) for a doubling in urinary and blood cadmium, respectively.

Our findings also remained consistent when we separately used the 24-hr urinary excretion of cadmium either at baseline or at follow-up as index of lifetime exposure and when we additionally adjusted for the time of day, at which participants collected urine for the measurement of HP and LP (data not shown).

## Discussion

Population-based studies from Belgium (Staessen et al. 1999), Sweden (Åkesson et al. 2006; Alfvén et al. 2000; Järup and Alfvén 2004), Japan (Honda et al. 2003), and China (Zhu et al. 2004) showed an association between osteoporosis and low-level environmental cadmium exposure. The interpretation of these findings was that cadmium-induced renal tubular damage (Buchet et al. 1990; Staessen et al. 1994) attenuated the calcium reabsorption in the nephron, resulting in hypercalciuria (Staessen et al. 1994) and demineralization of bones (Järup and Alfvén 2004; Staessen et al. 1999), particularly in menopausal women (Staessen et al. 1999). In keeping with experimental studies, the present study supports the interpretation that, in women, cadmium decreases bone density through a direct osteotoxic effect. Indeed, we found consistent associations between bio-markers of bone resorption, the urinary pyridinium crosslinks HP and LP, and biomarkers of lifetime and recent exposure to cadmium. In addition, serum PTH levels decreased with higher cadmium exposure, as might be expected when a toxic substance induces release of calcium from bone tissue. If the hypercalciuria in cadmium-exposed subjects were attributed entirely to excessive calcium loss in the renal tubules, one would expect instead an increase in PTH with higher exposure to compensate for the urinary calcium loss. A possible adaptive response favoring bone formation might occur, especially in postmenopausal women, with serum levels of bone-specific alkaline phosphatase activity and calcitonin correlating positively with cadmium exposure, as observed in the present study.

To our knowledge, only one previous population study has addressed the possible association between bone resorption and low-level cadmium exposure. In 820 Swedish women 53–64 years of age, Åkesson and colleagues (2006) measured forearm bone mineral density, calciotropic hormones, and the urinary LP concentration not standardized for creatinine. They assessed exposure to cadmium, not from the 24-hr urinary excretion, but from the concentration in fresh urine samples and blood. The median values were 4.6 nmol/L (0.52 μg/L) and 3.4 nmol/L (0.38 μg/L), respectively. In our current study, the corresponding concentrations in urine and blood were 5.2 nmol/L (0.58 μg/L) and 8.0 nmol/L (0.90 μg/L). In multivariate-adjusted analyses, Åkesson et al. (2006) reported inverse associations (*p* < 0.05) of bone density and serum PTH with the urinary cadmium concentration and a positive relation between urinary LP and cadmium. These associations persisted in never-smokers, who had the lowest, mainly dietary, cadmium exposure. For LP, there was a significant interaction between menopause and urinary cadmium. The associations with blood cadmium were not significant in the Åkesson et al. study, except for PTH.

Laboratory studies strongly support the epidemiologic evidence for a direct osteotoxic effect of cadmium. In experimental animals exposed to cadmium, bone demineralization begins early after the start of cadmium exposure, well before the onset of kidney damage (Wilson and Bhattacharyya 1997). In cultures of bone marrow cells, cadmium accelerated the differentiation of new osteoclasts from their progenitor cells and enhanced the activity of mature osteoclasts (Wilson et al. 1996). In female mice, bilateral ovariectomy enhanced the osteotoxicity of cadmium (Comelekoglu et al. 2007). However, at the molecular level, the effects of cadmium on bone tissue need further clarification. Cadmium stimulated bone resorption by the up-regulation of the production of prostaglandin E_2_ in osteoblasts through enhanced expression of phospholipase A_2_ and cyclooxygenase (Miyahara et al. 1992). Exposure of human osteoblast-like cells to cadmium also produced an increase in cas-pase-3 activity and nuclear changes characteristic of apoptosis, including marginalization and condensing of chromatin and DNA fragmentation (Coonse et al. 2007). Experiments in genetically engineered mice suggested that the effects of cadmium on bone tissue require c-*Src* (Regunathan et al. 2002) and might be mediated via a p38 mitogen-activated phosphokinase pathway (Regunathan et al. 2003), but are independent from c-*Fos* expression (Regunathan et al. 2002).

Our findings might have important implications for environmental policies, especially those designed to protect women’s health. Roughly, 200 million people worldwide suffer from osteoporosis (Reginster and Burlet 2006). In the United States, there are an estimated 44 million osteoporosis patients, of whom 30 million are women (Reginster and Burlet 2006). After menopause, osteoporosis occurs at an accelerated rate. The studies in Belgium (Staessen et al. 1999) and China (Zhu et al. 2004) demonstrated loss of bone mineral density in relation to cadmium exposure, which was more severe in women (Staessen et al. 1999; Zhu et al. 2004), particularly after the onset of menopause (Staessen et al. 1999). Itai-Itai disease in Japan, an advanced stage of cadmium-induced osteomalacia and osteoporosis combined with kidney disease, occurs almost exclusively in older women (Vahter et al. 2007). Women have a higher body burden of cadmium than men (Vahter et al. 2007). Low iron stores that are common during pregnancy and before menopause lead to an upregulation of the duodenal metal transporter, which has a high affinity for cadmium (Tallkvist et al. 2000; Vahter et al. 2007). In twin studies, the heritability of the blood cadmium concentration was 65% in nonsmoking women, but only 13% in nonsmoking men (Björkman et al. 2000). Finally, experimental studies showed stronger effects of cadmium on calciotropic hormones and on the metabolism of calcium and phosphate in female than in male rats (Brzóka and Moniuszko-Jakoniuk 2005).

The present study has limitations and strengths. Although our results were consistent after multiple adjustments and in sensitivity analyses, we cannot exclude residual confounding. We replicated the findings of Åkesson et al. (2006), albeit at higher exposure levels. However, the Swedish investigators measured LP and cadmium in concentration units on the same spot urine sample. We measured the crosslinks standardized to creatinine and 24-hr cadmium excretion on different samples, and we therefore excluded the possibility of a spurious association due to varying degrees of the concentration of urine in individual samples. Moreover, we also found significant dose–effect associations with blood cadmium, which with the exception of PTH was not the case in the Swedish study (Åkesson et al. 2006). Some experts consider LP as a more specific marker of bone resorption than HP (Robins 1983). However, both LP and HP originate from mature collagen. In most circumstances, bone collagen degradation is the major contributor to both crosslink compounds in urine, due to the low turnover rate of other tissues (McLaren et al. 1992). The urinary excretion of pyridinium crosslinks has a diurnal variation, with levels peaking in the morning (Schlemmer et al. 1992). Our results were consistent when we accounted for the starting time of the urine collection.

Cadmium is a ubiquitous and persistent environmental contaminant. In our study, zinc smelters began to emit cadmium into the atmosphere in 1888. The last zinc smelter shut down in 2002. Even though annual emissions dropped from 125,000 kg in 1950 to 130 kg in 1989, the historical pollution of the soil remains a source of exposure via food contamination and the inhalation of house dust (Hogervorst et al. 2007). In the United States, ecologic studies demonstrated cadmium pollution, not only close to industrial (Gale et al. 2004) or mining (Peplow and Edmonds 2004) settlements, but in agricultural (Schmitt et al. 2006) and coastal (Karouna-Renier et al. 2007) areas as well. Japanese women remain currently more exposed to cadmium than other rice-dependent populations in Asia and other parts of the world (Watanabe et al. 2004). Satarug and Moore (2004) predicted that the continuing mobilization of cadmium from once non-bioavailable geologic matrices into biologically accessible materials could gradually increase over the next 10–20 years and amplify the upward trend in osteoporosis in aging populations worldwide. Globally, in 2000, there were an estimated 9.0 million osteoporotic fractures (Johnell and Kanis 2006). These fractures caused the loss of 5.8 million disability-adjusted life-years, of which 51% occurred in Europe and the Americas (Johnell and Kanis 2006). Cadmium is also nephrotoxic (Staessen et al. 1994) and increases the risk of lung cancer (Nawrot et al. 2006). Regulators must realize that because of these health effects and the very long biological half-life of cadmium (Järup et al. 1998), exposure due to human activities is unacceptable.

In conclusion, cadmium is an osteotoxic pollutant that increases bone resorption. Even in the absence of cadmium-induced renal tubular dysfunction, low-level environmental exposure to cadmium increases calciuria with reactive changes in calciotropic hormones. The provisional tolerable daily intake of cadmium via food is currently 1 μg/kg per day (Nordberg 2004). The question arises whether, in the light of the present findings and the disability associated with osteoporotic fractures in aging populations (Johnell and Kanis 2006), regulators should not lower this threshold, particularly for women.

## Figures and Tables

**Table 1:** Characteristics of 294 women.

Characteristic	Premenopausal (*n* = 150)	Menopausal (*n* = 144)	*p*-Value
Anthropometrics			
Age (years)	39.1 ± 6.9	59.8 ± 8.8	< 0.0001
Body mass index (kg/m^2^)	24.9 ± 5.1	27.5 ± 5.8	< 0.0001
Follicle-stimulating hormone (U/L)	3.3 (0.1–18.5)	65.4 (30.0–137.6)	< 0.0001
Urinary creatinine (mmol/day)	10.0 ± 2.0	9.0 ± 2.2	< 0.0001
Biomarkers of exposure			
Blood cadmium at baseline (nmol/L)	10.2 (2.7–27.6)	11.7 (4.4–32.9)	0.085
Blood cadmium (nmol/L)	6.9 (1.8–23.1)	8.5 (3.6–24.9)	< 0.0001
Urinary cadmium at baseline (nmol/day)	6.3 (2.6–18.9)	11.8 (5.0–29.8)	< 0.0001
Urinary cadmium (nmol/day)	5.7 (2.0–17.5)	9.8 (4.0–25.1)	< 0.0001
Biomarkers of effect			
Proximal forearm density (g/cm^2^)	0.479 ± 0.052	0.405 ± 0.079	< 0.0001
Distal forearm density (g/cm^2^)	0.363 ± 0.057	0.308 ± 0.067	< 0.0001
HP (nmol/mmol creatinine)	37.9 (21.0–68.1)	46.8 (22.3–91.9)	< 0.0001
LP (nmol/mmol creatinine)	7.4 (3.6–17.6)	9.3 (2.6–20.5)	0.0004
Urinary calcium (mmol/day)	3.87 ± 2.03	4.31 ± 2.67	0.29
Serum total calcium (mmol/L)	2.31 ± 0.09	2.35 ± 0.11	0.058
Parathyroid hormone (pmol/L)	0.69 (0.07–2.70)	0.97 (0.07–3.14)	0.005
Calcitonin (nmol/L)	4.53 (2.67–7.96)	6.31 (3.71–10.5)	< 0.0001
Bone-alkaline phosphatase (U/L)	25.7 (10.0–64.0)	42.3 (18.0–103.0)	< 0.0001
Urinary retinol-binding protein (μg/day)	61.3 (24.9–143.1)	54.9 (24.2–164.8)	0.16
Lifestyle			
Physical activity (kcal/day)	562 (1–2,606)	345 (1–2,426)	0.12
Smoking (0, 1)	63 (42.0)	23 (16.0)	< 0.0001
Drinking (0, 1)	15 (10.0)	7 (4.9)	0.094
Middle or high socioeconomic status (0, 1)	46 (30.7)	5 (3.5)	< 0.0001
Intake of medications			
Diuretics (0, 1)	4 (2.7)	30 (20.8)	< 0.0001
Oral contraceptives (0, 1)	42 (28.0 )	NA	—
Hormonal substitution (0, 1)	NA	7 (4.9)	—

NA, not applicable. Values are arithmetic mean ± SD, geometric mean (5th–95th percentile interval), or number of women (%). Unless indicated otherwise, characteristics were measured at the time of the urine collection for crosslinks. To convert nanomoles of cadmium to micrograms, multiply by 0.11241; to convert millimoles of calcium to milligrams, multiply by 40.08; to convert picomoles of parathyroid hormone to nanograms, multiply by 9.428; to convert nanomoles calcitonin to micrograms, multiply by 3.4176.

**Table 2:** Characteristics of women across quartiles of the 24-hr urinary cadmium excretion.

	Quartiles of the distribution of 24-hr urinary cadmium				*p*-Value	
Characteristic	Low	Medium-low	Medium-high	High	For trend	For low vs. high
Limits of quartiles (nmol/day)	< 5.5	≥ 5.5– < 8.2	≥ 8.2– < 11.9	≥ 11.9		
No.	74	73	74	73		
Age (years)	37.4 ± 9.8	50.4 ± 12.3	52.0 ± 11.2	57.2 ± 10.0	< 0.0001	< 0.0001
Menopausal	9 (12.2)	36 (49.3)	41 (55.4)	58 (79.5)	< 0.0001	< 0.0001
Parity [no. (range)]	2 (0–5)	2 (0–12)	2 (0–8)	2 (0–11)	0.47	0.19
Used food supplements^a^	8 (10.8)	11 (15.1)	8 (10.8)	7 (9.6)	0.74	0.24
HP (nmol/mmol creatinine)	37.6 (22.6–73.4)	41.6 (20.7–74.2)	42.3 (22.8–86.0)	47.5 (23.6–89.5)	0.028	0.003
LP (nmol/mmol creatinine)	7.5 (4.0–15.9)	8.6 (3.5–16.3)	8.2 (2.5–20.0)	9.1 (2.2–19.8)	0.37	0.095
Urinary calcium (mmol/day)	3.09 ± 4.18	3.76 ± 3.47	3.70 ± 3.53	5.83 ± 3.74	0.0002	0.0002
Serum calcium (mmol/L)	2.32 ± 0.12	2.35 ± 0.10	2.33 ± 0.10	2.32 ± 0.11	0.30	0.79
Bone-alkaline phosphatase (U/L)	33.0 (16.4–74.1)	29.7 (9.5–65.8)	35.1 (14.9–87.1)	33.9 (13.5–94.6)	0.32	0.81
Parathyroid hormone (pmol/L)	0.96 (0.11–2.52)	0.91 (0.07–3.49)	0.81 (0.07–3.33)	0.63 (0.07–2.35)	0.13	0.046
Calcitonin (nmol/L)	5.15 (2.82–8.61)	5.22 (2.74–9.17)	5.39 (2.98–9.12)	5.56 (3.28–10.4)	0.64	0.26

Values are arithmetic mean ± SD, geometric mean (5th–95th percentile interval), or number of women (%).

a Calcium and/or vitamin D.

**Table 3:** Independent associations of forearm bone density and effect biomarkers in urine with lifetime exposure as reflected by 24-hr cadmium excretion.

	Forearm bone density		Biomarkers in urine		
	Proximal (g/cm^2^)	Distal (g/cm^2^)	HP (log nmol/mmol creatinine)	LP (log nmol/mmol creatinine)	Calcium (mmol/day)
*R*^2^	0.454	0.371	0.206	0.128	0.066
Intercept	0.278	0.161	1.990	1.253	0.399
Partial regression coefficients (± SE)					
Urinary cadmium (log nmol/day)	–0.030 ± 0.016*	NS	0.116 ± 0.044^#^	0.096 ± 0.063*	2.568 ± 0.846^#^
Menopause (0, 1)	NS	NS	NS	NS	NS
Urinary cadmium × menopause	NS	–0.041 ± 0.015^#^	NS	NS	NS
Age (years × 10^–1^)	0.087 ± 0.012##	0.070 ± 0.020##	–0.267 ± 0.059##	–0.267 ± 0.084^#^	NS
Age squared (years^2^ × 10^–3^)	–0.115 ± 0.019##	0.091 ± 0.19##	0.286 ± 0.055##	0.298 ± 0.078^#^	NS
Body mass index (kg/m^2^ × 10^–1^)	NS	NS	0.069 ± 0.019##	0.048 ± 0.026*	NS
γ-glutamyltransferase (log U/L)	0.041 ± 0.013^#^	0.054 ± 0.013##	–0.064 ± 0.038*	NS	NS
Use of diuretics (0, 1)	NS	NS	NS	–0.143 ± 0.047^#^	–1.798 ± 0.669^#^
Physical activity (log kcal/day × 10^–1^)	0.050 ± 0.029*	NS	NS	NS	NS

Significance of the partial regression coefficients: NS, not significant;

* 0.1 < *p* < 0.05;

## *p* ≤ 0.001. Socioeconomic position and the 24-hr excretion of retinol-binding protein did not enter any model.

**Table 4:** Independent associations of effect biomarkers in serum with lifetime exposure as reflected by 24-hr cadmium excretion.

	Calcium (mmol/L)	Parathyroid hormone (log pmol/L)	Calcitonin (log nmol/L)	Bone-specific alkaline phosphatase (log U/L)
*R*^2^	0.045	0.083	0.388	0.301
Intercept	2.376	–0.814	1.226	1.921
Partial regression coefficients (± SE)				
Urinary cadmium (log nmol/day)	NS	–0.224 ± 0.121*	NS	NS
Menopause (0, 1)	0.035 ± 0.014**	NS	0.096 ± 0.039**	1.151 ± 0.061**
Urinary cadmium × menopause	NS	NS	0.066 ± 0.036*	0.140 ± 0.061**
Age (years × 10^–1^)	NS	NS	–0.119 ± 0.047**	NS
Age squared (years^2^ × 10^–3^)	NS	NS	0.120 ± 0.044#	NS
Body mass index (kg/m^2^ × 10^–1^)	–0.0239 ± 0.011**	0.086 ± 0.048*	–0.033 ± 0.015**	NS
γ-glutamyltransferase (log U/L)	NS	NS	–0.179 ± 0.030##	–0.312 ± 0.052##
Smoking (0, 1)	NS	–0.104 ± 0.061*	NS	NS
Physical activity (log kcal/day × 10^–1^)	NS	NS	NS	–0.308 ± 0.011#
Use of diuretics (0, 1)	NS	NS	–0.067 ± 0.026**	–0.119 ± 0.044#
Intake of female hormones (0,1)	NS	NS	–0.074 ± 0.021##	NS

Significance of the partial regression coefficients: NS, not significant;

* 0.1 < *p* < 0.05;

** *p* ≤ 0.05;

# *p* ≤ 0.01;

## *p* ≤ 0.001. Socioeconomic position and the 24-hr excretion of retinol-binding protein did not enter any model.

**Table 5:** Independent associations of forearm bone density and effect biomarkers in urine with current exposure as reflected by blood cadmium.

	Forearm bone density		Biomarkers in urine		
	Proximal (g/cm^2^)	Distal (g/cm^2^)	HP (log nmol/mmol creatinine)	LP (log nmol/mmol creatinine)	Calcium (mmol/day)
*R*^2^	0.446	0.358	0.216	0.139	0.042
Intercept	0.300	0.168	1.847	1.193	1.411
Partial regression coefficients (± SE)					
Blood cadmium (log nmol/L)	NS	NS	0.100 ± 0.030##	0.100 ± 0.043**	NS
Menopause (0, 1)	NS	NS	NS	NS	NS
Blood cadmium × menopause	NS	–0.029 ± 0.014**	NS	NS	NS

Significance of the partial regression coefficients: NS, not significant;

** *p* < 0.05;

## *p* < 0.001. All models were adjusted for the same covariates as in Table 3.

**Table 6:** Independent associations of effect biomarkers in serum with current exposure as reflected by blood cadmium.

	Calcium (mmol/L)	Parathyroid hormone (log pmol/L)	Calcitonin (log nmol/L)	Bone-specific alkaline phosphatase (log U/L)
*R*^2^	0.046	0.068	0.397	0.300
Intercept	2.383	–0.756	1.186	1.933
Partial regression coefficients (± SE)				
Blood cadmium (log nmol/L)	NS	–0.136 ± 0.082*	0.078 ± 0.029#	NS
Menopause (0, 1)	0.034 ± 0.013#	NS	0.152 ± 0.025#	0.187 ± 0.056##
Blood cadmium × menopause	NS	NS	NS	0.098 ± 0.058*

Significance of the partial regression coefficients: NS, not significant;

* 0.1 < *p* < 0.05;

# *p* ≤ 0.01;

## *p* ≤ 0.001. All models were adjusted for the same covariates as in Table 4.

## References

[b1-ehp0116-000777] Åkesson A, Bjellerup P, Lundh T, Lidfeldt J, Nerbrand C, Samsioe G (2006). Cadmium-induced effects on bone in a population-based study of women. Environ Health Perspect.

[b2-ehp0116-000777] Alfvén T, Elinder CG, Carlsson MD, Grubb A, Hellström L, Presson B (2000). Low level cadmium exposure and osteoporosis. J Bone Miner Res.

[b3-ehp0116-000777] Bartels H, Böhmer M (1971). Ein Mikromethode zur Kreatinin Bestimmung [in German]. Clin Chem Acta.

[b4-ehp0116-000777] Björkman L, Vahter M, Pedersen NL (2000). Both the environment and genes are important for concentrations of cadmium and lead in blood. Environ Health Perspect.

[b5-ehp0116-000777] Black D, Duncan A, Robins SP (1998). Quantitative analysis of the pyridinium crosslinks of collagen in urine using ion-paired reversed-phase high-performance liquid chromatography. Anal Biochem.

[b6-ehp0116-000777] Bouillon R, Coopmans W, Degroote DEH, Radoux D, Eliard PH (1990). Immunoradiometric assay of parathyrin with polyclonal and monoclonal region specific antibodies. Clin Chem.

[b7-ehp0116-000777] Brzóka MM, Moniuszko-Jakoniuk J (2005). Effect of low-level lifetime exposure to cadmium on calciotropic hormones in aged femal rats. Arch Toxicol.

[b8-ehp0116-000777] Buchet JP, Lauwerys R, Roels H, Bernard A, Bruaux P, Claeys F (1990). Renal effects of cadmium body burden of the general population. Lancet.

[b9-ehp0116-000777] Choudhury H, Harvey T, Thayer WC, Lockwood TF, Stiteler WM, Goodrum PE (2001). Urinary cadmium elevation as a biomarker of exposure for evaluating a cadmium dietary exposure biokinetic model. J Toxicol Environ Health Part A.

[b10-ehp0116-000777] Comelekoglu U, Yalin S, Bagis S, Ogenler O, Sahin NO, Yildir A (2007). Low-exposure cadmium is more toxic on osteoporotic rat femoral bone: mechanical, biochemical, and histopathological evaluation. Ecotoxicol Environ Saf.

[b11-ehp0116-000777] Coonse KG, Coonts AJ, Morrison EV, Heggland SJ (2007). Cadmium induces apoptosis in the human osteoblast-like cell line Saos-2. J Toxicol Environ Health.

[b12-ehp0116-000777] Gale NL, Adams CD, Wixson BG, Loftin KA, Huang YW (2004). Lead, zinc, copper, and cadmium in fish and sediments from the Big River and Flat River Creek of Missouri’s old lead belt. Environ Geochem Health.

[b13-ehp0116-000777] Hogervorst J, Plusquin M, Vangronsveld J, Nawrot T, Cuypers A, Van Hecke E (2007). House dust as possible route of environmental exposure to cadmium and lead in the adult general population. Environ Res.

[b14-ehp0116-000777] Honda R, Tsuritani I, Noborisaka Y, Suzuki H, Ishizaki M, Yamada Y (2003). Urinary cadmium excretion is correlated with calcaneal bone mass in Japanese women living in an urban area. Environ Res.

[b15-ehp0116-000777] Järup L, Alfvén T (2004). Low level cadmium exposure, renal and bone effects—the OSCAR study. Biometals.

[b16-ehp0116-000777] Järup L, Berglund M, Elinder CG, Nordberg G, Vahter M (1998). Health effects of cadmium exposure–a review of the literature and a risk estimate. Scand J Work Environ Health.

[b17-ehp0116-000777] Johnell O, Kanis JA (2006). An estimate of the worldwide prevalence and disability associated with osteoporotic fractures. Osteoporosis Int.

[b18-ehp0116-000777] Karouna-Renier NK, Snyder RA, Allison JG, Wagner MG, Ranga RK (2007). Accumulation of organic and inorganic contaminants in shellfish collected in estuarine waters near Pensacola, Florida: contamination profiles and risks to human consumers. Environ Pollution.

[b19-ehp0116-000777] Lauwerys R, Amery A, Bernard A, Bruaux P, Buchet JP, Claeys F (1990). Health effects of environmental exposure to cadmium: objectives, design and organization of the Cadmibel study: a cross-sectional morbidity study carried out in Belgium from 1985 to 1989. Environ Health Perspect.

[b20-ehp0116-000777] Lauwerys RR, Hoet P (2001). Biological monitoring of exposure to inorganic and organometallic substances. Cadmium. Industrial Chemical Exposure. Guidelines for Biochemical Monitoring.

[b21-ehp0116-000777] McLaren AM, Hordon LD, Bird HA, Robins SP (1992). Urinary excretion of pyridinium crosslinks of collagen in patients with osteoporosis and the effects of bone fracture. Ann Rheum Dis.

[b22-ehp0116-000777] Miyahara T, Takata M, Mori-Uchi S, Miyata M, Nagai M, Sugure A (1992). Stimulative effects of cadmium on bone resorption in neonatal parietal bone resorption. Toxicology.

[b23-ehp0116-000777] Nawrot T, Plusquin M, Hogervorst J, Roels HA, Celis H, Thijs L (2006). Environmental exposure to cadmium and risk of cancer: a prospective population-based study. Lancet Oncol.

[b24-ehp0116-000777] Nordberg M (2004). Environmental exposure and preventive measures in Sweden and EU. Biometals.

[b25-ehp0116-000777] Peplow D, Edmonds R (2004). Health risks associated with contamination of groundwater by abandoned mines near Twisp in Okanogan County, Washington, USA. Environ Geochem Health.

[b26-ehp0116-000777] Reginster JY, Burlet N (2006). Osteoporosis: a still increasing prevalence. Bone.

[b27-ehp0116-000777] Regunathan A, Cerny EA, Villarreal J, Bhattacharyya MH (2002). Role of *fos* and *src* in cadmium-induced decreases in bone mineral content in mice. Toxicol Appl Pharmacol.

[b28-ehp0116-000777] Regunathan A, Glesne DA, Wilson AK, Song J, Nicolae D, Flores T (2003). Microarray analysis of changes in bone cell gene expression early after cadmium gavage in mice. Toxicol Appl Pharmacol.

[b29-ehp0116-000777] Robins SP (1983). Cross-linking of collagen. Isolation, structural characterization and glycosylation of pyridinoline. Biochem J.

[b30-ehp0116-000777] Satarug S, Moore MR (2004). Adverse health effects of chronic exposure to low-level cadmium in foodstuffs and cigarette smoke. Environ Health Perspect.

[b31-ehp0116-000777] Schlemmer A, Hassager C, Jensen SB, Christiansen C (1992). Marked diurnal variation in urinary excretion of pyridinium cross-links in premenopausal women. J Clin Endocrinol Metab.

[b32-ehp0116-000777] Schmitt CJ, Brumbaugh WG, Linder GL, Hinck JE (2006). A screening-level assessment of lead, cadmium, and zinc in fish and crayfish from Northeastern Oklahoma, USA. Environ Geochem Health.

[b33-ehp0116-000777] Staessen J, Amery A, Bernard A, Bruaux P, Buchet JP, Claeys F (1991). Effects of exposure to cadmium on calcium metabolism: a population study. Br J Ind Med.

[b34-ehp0116-000777] Staessen JA, Lauwerys RR, Ide G, Roels HA, Vyncke G, Amery A (1994). Renal function and historical environmental cadmium pollution from zinc smelters. Lancet.

[b35-ehp0116-000777] Staessen JA, Roels HA, Emelianov D, Kuznetsova T, Thijs L, Vangronsveld J (1999). Environmental exposure to cadmium, forearm bone density, and risk of fractures: prospective population study. Lancet.

[b36-ehp0116-000777] Tallkvist J, Bowlus CL, Lönnerdal B (2000). *DMT1* gene expression and cadmium absorption in human absorptive enterocytes. Toxicol Lett.

[b37-ehp0116-000777] Uebelhart D, Gineyts E, Chaouy MC, Delmas PD (1990). Urinary excretion of pyridinium crosslinks: a new marker of bone resorption in metabolic bone disease. Bone Miner.

[b38-ehp0116-000777] Vahter M, Åkesson A, Lidén C, Ceccatelli S, Berglund M (2007). Gender differences in the disposition and toxicity of metals. Environ Res.

[b39-ehp0116-000777] Wang C, Brown S, Bhattacharyya MH (1994). Effect of cadmium on bone calcium and ^45^Ca in mouse dams on a calcium-deficient diet: evidence of Itai-Itai-like syndrome. Toxicol Appl Pharmacol.

[b40-ehp0116-000777] Watanabe T, Zhang ZW, Moon CS, Shimbo S, Nakatsuka H, Matsuda-Inoguchi N (2004). Cadmium exposure of women in general populations in Japan during 1991–1997 compared with 1977–1981. Int Arch Occup Environ Health.

[b41-ehp0116-000777] Wilson AK, Bhattacharyya MH (1997). Effects of cadmium on bone: an in vivo model for the early response. Toxicol Appl Pharmacol.

[b42-ehp0116-000777] Wilson AK, Cerny EA, Smith BD, Wagh A, Bhattacharyya MH (1996). Effects of cadmium on osteoclast formation and activity *in vitro*. Toxicol Appl Pharm.

[b43-ehp0116-000777] Zhu G, Wang H, Shi Y, Weng S, Jin T, Kong Q (2004). Environmental cadmium exposure and forearm bone density. Biometals.

